# Genome-Wide Screening and Characterization of Non-Coding RNAs in *Coffea canephora*

**DOI:** 10.3390/ncrna6030039

**Published:** 2020-09-11

**Authors:** Samara M. C. Lemos, Luiz F. C. Fonçatti, Romain Guyot, Alexandre R. Paschoal, Douglas S. Domingues

**Affiliations:** 1 Department of Computer Science, Federal University of Technology-Parana, Cornelio Procopio 86300-000, Brazil; samaramclemos@gmail.com (S.M.C.L.); luifon@alunos.utfpr.edu.br (L.F.C.F.); paschoal@utfpr.edu.br (A.R.P.); 2 Group of Genomics and Transcriptomes in Plants, São Paulo State University, Rio Claro, Sao Paulo 13506-900, Brazil; 3 Institut de Recherche pour le Développement (IRD), University Montpellier, 34394 Montpellier, France; romain.guyot@ird.fr

**Keywords:** coffee, plant ncRNAs, curated annotation, genome-wide, bioinformatics, in silico

## Abstract

*Coffea canephora* grains are highly traded commodities worldwide. Non-coding RNAs (ncRNAs) are transcriptional products involved in genome regulation, environmental responses, and plant development. There is not an extensive genome-wide analysis that uncovers the ncRNA portion of the *C. canephora* genome. This study aimed to provide a curated characterization of six ncRNA classes in the *Coffea canephora* genome. For this purpose, we employed a combination of similarity-based and structural-based computational approaches with stringent curation. Candidate ncRNA loci had expression evidence analyzed using sRNA-seq libraries. We identified 7455 ncRNA loci (6976 with transcriptional evidence) in the *C. canephora* genome. This comprised of total 115 snRNAs, 1031 snoRNAs, 92 miRNA precursors, 602 tRNAs, 72 rRNAs, and 5064 lncRNAs. For miRNAs, we identified 159 putative high-confidence targets. This study was the most extensive genomic catalog of curated ncRNAs in the Coffea genus. This data might help elaborating more robust hypotheses in future comparative genomic studies as well as gene regulation and genome dynamics, helping to understand the molecular basis of domestication, environmental adaptation, resistance to pests and diseases, and coffee productivity.

## 1. Introduction

Non-coding RNAs (ncRNAs) are functional regulatory molecules that mediate cellular processes, including chromatin remodeling, transcription, post-transcriptional modifications, and signal transduction [1,2]. They can be roughly divided into two categories: housekeeping and regulatory ncRNAs [3]. Housekeeping ncRNAs, such as ribosomal RNAs (rRNAs), transfer RNAs (tRNAs), small nuclear RNAs (snRNAs), and small nucleolar RNAs (snoRNAs), are responsible for basic cellular functions. Regulatory ncRNAs, such as microRNAs (miRNAs), small interfering RNAs (siRNAs), and long non-coding RNAs (lncRNAs), are capable of moving between cells, performing cell-to-cell signaling in development or physiology, and mediating epigenetic inheritance [4].

In plants, most studies usually identify a single class of ncRNAs, such as miRNAs or long non-coding RNAs [4,5,6,7,8]. Although several studies have identified miRNAs in plants [9,10,11], the identification has not always been performed in the most reliable way [12]. Criteria to identify and annotate miRNAs have been in constant improvement over time [12,13,14,15].

Coffee is one of the world’s most popular beverages, and 80% of it is produced by 25 million smallholders. Around 125 million people worldwide depend on coffee for their livelihoods. *Coffea canephora* is one of the most important agricultural commodities, corresponding to nearly 40% of the world coffee production [16] and the first coffee species with a publicly available genome annotation [17,18]. The *Coffea canephora* genome annotation has identified 25,574 protein-coding genes, and approximately 50% of the genome is composed of transposable elements [18]. The repertoire of annotated non-coding RNAs in that study [18] is restricted to microRNAs (92 precursors). To date, information about ncRNAs for *Coffea canephora* is not clearly organized.

A total of seven studies have predicted miRNAs in *C. canephora* using distinct approaches. Five studies have focused only on prediction [18,19,20,21,22]. Two studies have used small RNA sequencing to confirm the transcriptional activity of miRNAs [23,24]. There is only one study [22] that has focused on a comprehensive analysis of the *C. canephora* sequenced genome to annotate miRNAs, but this study has not taken account of recent changes in miRNA annotation rules in plants. For example, the criteria concerning expression analysis and the curation of precursors have become more strict, and they have an impact on defining high-quality prediction of miRNA loci [12,15].

In this context, there is a gap in a highly accurate ncRNAs annotation for coffee. For that, we provided the most extensive and organized ncRNA loci catalog of the *Coffea canephora* genome. We characterized snRNAs, snoRNAs, miRNAs, tRNAs, rRNAs, lncRNAs and performed a manual standardization of the previously identified microRNAs in the species.

## 2. Results

### 2.1. Non-Coding RNA Loci Overview

**Figure 1:**
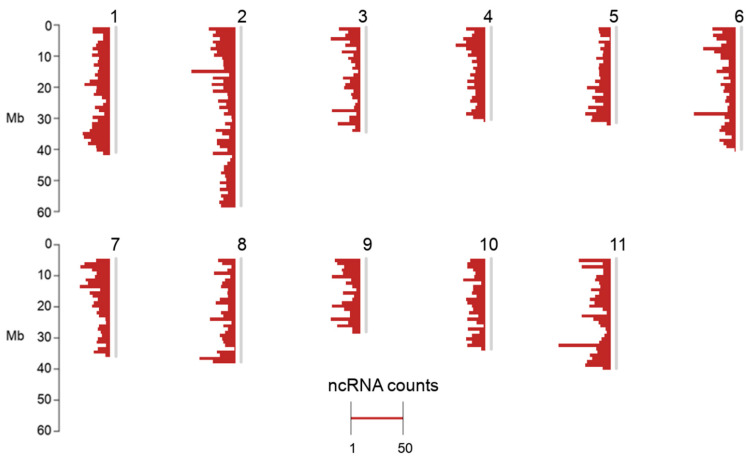
Distribution of ncRNAs in *Coffea canephora* genome.

A total of 6976 expressed ncRNA loci were identified in the *C. canephora* genome. About 64% of the *C. canephora* genomic sequence was assembled on 11 chromosomes [18]. Assembled contigs that were not assigned to any chromosome were grouped arbitrarily into a pseudomolecule named “Chromosome 0” [18]. All results from the pseudomolecule “Chromosome 0” are presented separately in Supplementary File S3. For practical reasons, we considered only assembled chromosomes for overall statistics. Among the assembled chromosomes, 3966 expressed ncRNA loci were identified. Chromosome 2 had the highest number of loci (626 ncRNA loci), and chromosome 3 showed the lowest number of ncRNA loci, with 203 (Table S1, Supplementary File S1). The distribution of ncRNAs among chromosomes is detailed in Table S2, Supplementary File S1, and Figure 1 using chromPlot [25]. We did not identify any preferential location of ncRNA loci.

Ninety-three snRNAs, 778 snoRNAs, 427 tRNAs, 51 rRNAs, 88 miRNAs, and 2529 lncRNAs were determined as high-confidence ncRNAs. The average length of small ncRNAs (snRNAs, snoRNAs, tRNAs, rRNAs, and miRNA precursors) was 126 nt, smaller than the mean length of protein-coding genes CDS (1206 nt) and introns (483 nt) [18]. The number of ncRNA loci in *Coffea canephora* was comparable to other curated ncRNA annotations in other plant species (Table 1). In summary, we noticed that tRNA, rRNA, and snRNA loci were in the range of most analyzed species (Table 1), even considering variation. For miRNA, we expected this result since we used a more stringent criterium of miRNA annotation, following several steps of filtering to avoid high false-positives. In the case of snoRNAs, we used a novel tool (SnoReport 2), which seemed to explain differences in discovery. Finally, we had applied the most updated pipeline for lncRNA prediction, which brought us a close number against other species except *Clonorchis sinensis*. Although *C. canephora* genome size is similar to the size of *Proteus vulgaris* and *Berberis vulgaris* genomes, evolutionary differences and applied methodologies should be taken into account before any comparison.

### 2.2. Small Nuclear RNAs

snRNAs bind to specific proteins and compose the spliceosome, a large ribonucleoprotein complex responsible for the alternative splicing process [26], which is crucial since it increases diversity. In plants, the extension of the alternative splicing process can reach over 60% of intron-containing genes [27].

We identified 93 high-confidence snRNA regions, ranging from 90 to 199 nucleotides and belonging to eight spliceosomal U snRNA families (Table S8, Supplementary File S2). There are nine spliceosomal U snRNAs: (U1, U2, U4, U4 atac, U5, U6, U6 atac, U11, and U12) [26]. The U snRNAs loci identified in the *Coffea canephora* genome were from families U1 (21), U2 (16), U4 (6), U5 (9), U6 (36), U6atac (2), U11 (2), and U12 (1). U4 atac snRNAs were not identified in *C. canephora*. These are not usually found in plants, and they have been only identified in *Arabidopsis thaliana* and *Beta vulgaris* [28].

*C. canephora* snRNA genes were distributed mostly on chromosome 8 with 12 snRNAs, followed by chromosome 2 with 11 loci and chromosome 1 with 10 loci (Table S1, Supplementary File S1). A total of three loci did not have transcriptional evidence (Table S7, Supplementary File S2).

Twenty-two high-confidence snRNA regions were identified in the pseudomolecule “Chromosome 0”. The snRNAs that were not assigned to any chromosome had sizes ranging from 92 to 203 nucleotides and belonged to five spliceosomal U snRNA families: U1 (4), U2 (7), U4 (3), U5 (2), and U6 (6) (Table S19, Supplementary File S3).

### 2.3. Small Nucleolar RNAs

snoRNAs accumulate in the nucleolus and play a role in site-specific RNA modifications [29]. C/D box snoRNAs have one or more C/D box motifs in their sequence and form complex ribonucleoproteins (snoRNPs) that guides 2′-O-ribose methylation of ribonucleotides like pre-rRNAs [26]. H/ACA box snoRNAs have H/ACA box motifs in their sequence and compose small nucleolar ribonucleoproteins (snoRNPs) that guide the pseudouridylation of specific nucleotides [26].

We identified 778 high-confident snoRNA loci, ranging from 71 to 197 nucleotides in the *C. canephora* genome (Table S9, Supplementary File S2). Most loci had C/D box motifs (662), and 116 loci had H/ACA motifs. The *C. canephora* snoRNAs were mostly on chromosome 2, with 104 loci, followed by chromosome 4 with 85 loci and chromosome 11 with 84 loci (Table S1 and Supplementary File S1).

Two hundred and fifty-three high-confidence snoRNA regions were identified in the pseudomolecule “Chromosome 0”. The snoRNAs that were not assigned to any chromosome had sizes ranging from 76 to 212 nucleotides. Two hundred and forty-five loci had C/D box motifs, and eight loci had H/ACA motifs (Table S20, Supplementary File S3).

Some snoRNAs were organized in clusters: we found 53 genes in 22 clusters (Table S2, Supplementary File S1). snoRNA clusters are encoded or positioned closely in the same chromosomal region [29]. They have been identified in other plants: 43 in *A. thaliana* and 68 in *Oryza sativa* [30,31]. There are at least 110 snoRNA conserved families in the plant kingdom [29]. In our data, we found 56 families conserved in plants (Table S3, Supplementary File S1). The most identified C/D snoRNA family was R71 with 28 loci, also identified in *A. thaliana* and *Brassica rapa* [29]. The most identified H/ACA snoRNA family was R74 with six loci (Table S3, Supplementary File S1), also identified in *Vitis vinifera, C. sinensis,* and other species [29]. We observed seven loci with no transcriptional evidence (Table S7, Supplementary File S2).

### 2.4. Transfer RNAs

Transfer RNAs (tRNAs) carry amino acids and act in protein translation machinery [32]. We identified 427 high-confidence tRNA loci, ranging from 70 to 92 nucleotides (Table S10, Supplementary File S2). Most tRNA genes were on chromosome 2 with 91 genes, followed by chromosome 7 with 55 genes (Table S1, Supplementary File S1). tRNA^Met^ was the most frequent tRNA gene, with 61 loci. The less frequent was tRNA^His^ with 15 genes. tRNA genes copy numbers could vary among species (Table S4, Supplementary File S1).

One hundred and seventy-five high-confidence tRNA regions were identified in the pseudomolecule “Chromosome 0”. The tRNAs that were not assigned to any chromosome had sizes ranging from 70 to 92 nucleotides (Table S21, Supplementary File S3).

For each of the 20 essential amino acids, there are at least five possible anti-codons/isoacceptors totalizing 61 in the genetic code [32]. In our analyses, we identified 54 anti-codons/isoacceptors. Similar analyses in eudicots and monocots have presented anti-codons/isoacceptors, ranging from 45 to 52 [32,33]. tRNALeu was the most abundant with six isoacceptors [AAG (6) CAA (16) CAG (7) TAA (5) TAG (7), GAG (1)]; all isoacceptors are organized in Table S5, Supplementary File S1. In counterpart, tRNAAsp, tRNAMet, tRNACys, tRNAPhe, tRNATrp, and tRNATyr had only one isoacceptor.

Using tRNAscan-SE [34], one putative gene for tRNASeC was also identified, which is responsible for selenocysteine biosynthesis. Although higher plants do not possess selenoproteins due to the absence of the selenocysteine insertion machinery, tRNASeC genes were searched in the *C. canephora* genome using SecMarker [35], and it was concluded that this locus was a false-positive. Previous studies have already reported that tRNAscan-SE can identify false positives for tRNASeC in plants [36].

### 2.5. Ribosomal RNAs

Ribosomal RNAs (rRNAs) associate with a set of proteins to form a small or a large subunit of ribosomes [37]. Fifty-one high-confidence rRNA loci were identified, ranging from 82 to 1275 nucleotides, after merging results and excluding redundancies (Table S11, Supplementary File S2). A total of 14 loci did not have transcriptional evidence (Table S7, Supplementary File S2). Units 5S (34), 5.8S (8), two fragments from a large ribosomal subunit, five fragments from a small ribosomal subunit were identified, while two were indeterminate.

Twenty-one high-confidence rRNA regions were identified in the pseudomolecule “Chromosome 0”. The rRNAs that were not assigned to any chromosome had sizes ranging from 76 to 3992 nucleotides (Table S22, Supplementary File S3).

Ribosomal RNA genes were highly conserved and organized as repetitive units, supporting the different number of rRNA genes identified in chromosome 2 and chromosome 11 (Table S1, Supplementary File S1). Besides, their copy numbers might vary among species: *C. canephora* had a larger number of rRNA genes when compared to *Daucus carota* [38] and a smaller number when compared to 149 from *Phaseoulus vulgaris* [39], 232 in *Beta vulgaris* [28], and 2860 from *Citrus sinensis* [40]. It is important to consider that different methods have been applied among these studies, and completeness of genomic assemblies have a crucial impact on finding those loci.

### 2.6. MicroRNAs Prediction

Recent studies have pointed out a large number of false-positive miRNAs identified in plants [12,15]. For example, in *Citrus sinensis*, only 98 of the 227 miRNA loci previously described have fulfilled all annotation criteria [12]. In our miRNA prediction, a list of 109 candidate regions was obtained, and a filter was applied in them to exclude overlapping cases. One hundred and eight miRNA precursors were evaluated using miRdup [41], and 216 putative mature miRNAs were predicted. Axtell and Meyers’s (2018) criteria were also applied in these candidates, and 11 precursors were selected (Table S12, Supplementary File S2).

The set of miRNAs consisted of 10 precursors in the assembled chromosomes plus 1 precursor that were not assigned to any chromosome (“Chromosome 0”), each one with a pair of mature sequences and belonging to five conserved plant miRNA families. The 22 mature miRNAs had sizes ranging between 21 and 22 nt and were identified in three independent sRNA libraries (Table S13, Supplementary File S2).

Comparing our results with previously identified *C. canephora* miRNAs [18,19,20,21,22,23,24], we identified seven new precursors and 18 new mature miRNAs for *C. canephora* (Tables S12 and S13, Supplementary File S2, Supplementary File S3).

### 2.7. MicroRNAs Curation

In order to have a highly curated and standardized miRNA atlas for *C. canephora*, we applied Axtell and Meyers (2018) criteria in 495 putative miRNA precursors and 672 putative mature miRNAs from previous studies [18,19,20,21,22,23,24].

In our analysis, we found 114 redundant sequences in the 495 retrieved precursors. Two hundred and eighty-two precursors had divergent mature sequences or did not have expression, 14 precursors had no hairpin or were longer than 300 nt, seven precursors presented only one mature sequence, and 186 failed in more than one criterion (Table S6, Supplementary File S1). Some studies have identified only one mature miRNA sequence, and those sequences were excluded from our analysis due to the “only one mature sequence” criterion. In Guedes et al. (2018) study, miR-398 and miR-408 were predicted with only one mature miRNA sequence but had their sequences validated by qPCR experiments, so we considered the results.

Therefore, we considered that 60 precursors and 120 mature sequences matched the curation criteria in published data (Supplementary File S4). In summary, the *C. canephora* set of high-standard miRNAs was composed of 72 precursors and 145 mature miRNAs belonging to 27 families (Tables S12 and S13, Supplementary File S2). Most of the identified miRNA families are variants of miR-156, miR-159, miR-162, miR-164, miR-166, miR-167, miR-168, miR-169, miR-171, miR-172, miR-319, miR-390, miR-393, miR-394, miR-395, miR-396, miR-397, miR-398, miR-399, miR-403, miR-408, miR-477, miR-482, miR-530, and miR-2111, all conserved in plants [15,42]. Families miR-171 and miR-399 were the most numerous, with 11 and six loci, respectively. miR-171 family is upregulated in *A. thaliana* under nitrogen deprivation and in *Sorghum bicolor* in zinc deprivation [43]. In *Coffea arabica*, mir-171 is also upregulated under short-term nitrogen deprivation [44]. Family miR-399 is well known to be involved in phosphate homeostasis [45].

### 2.8. Phylogenetic Analysis of MicroRNAs

**Figure 2:**
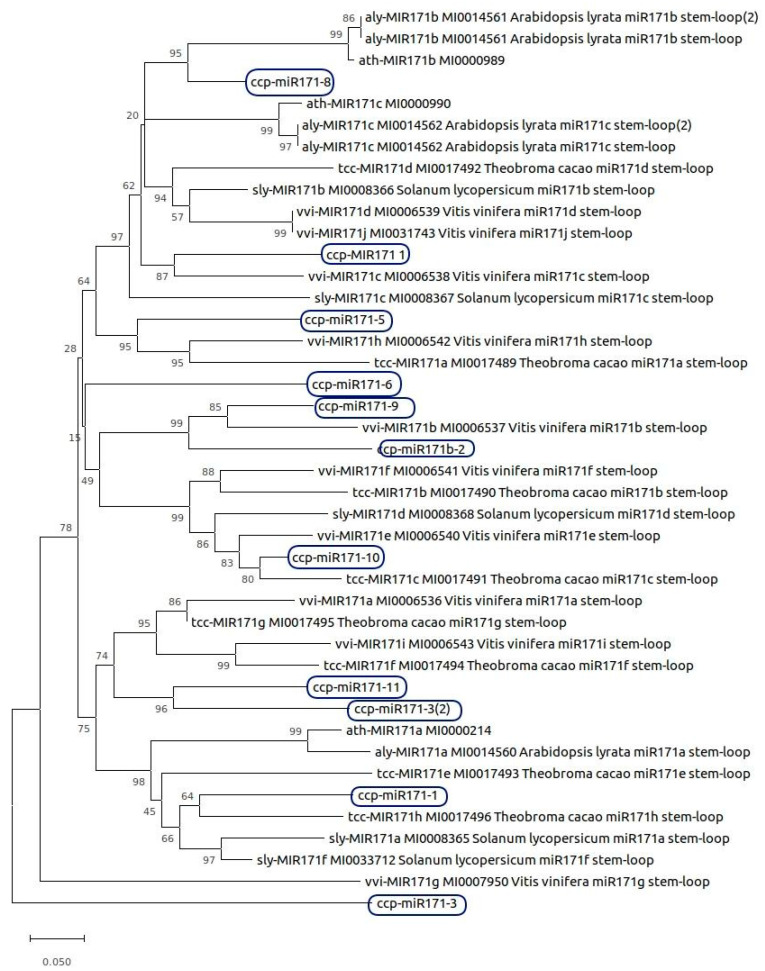
Phylogenetic analysis of miRNA family miR-171. The highlighted precursors showed high conservation among a total of 42 sequences, including their orthologs.

Among the 60 precursors, miRNAs from family miR-171 were the most numerous identified; therefore, we investigated their conservation with orthologs in *Arabidopsis thaliana*, *Arabidopsis lyrata, Solanum lycopersicum, Theobroma cacao,* and *Vitis vinifera* (Figure 2).

### 2.9. MicroRNA Targets Prediction

Understanding miRNA-made regulation is possible through the identification of their target genes [43]. We identified 10,714 potential targets for 176 miRNAs, with an average of 60.9 targets per miRNA (Table S14, Supplementary File S2). Family miR-396 had a larger number of targets (>9 targets for each family member), a fact also observed in *Manihot esculenta* [46] and *Jatropha curcas* [47].

Annotation data of potential targets were imported from Plaza 4.0 database [48]. A total of 157 high-confidence targets, based on the expectation score, were classified according to their gene ontology classes (GO terms), InterPro results, and similarity with *A. thaliana* genes (Tables S14–S16, Supplementary File S2). A total of 605 GO terms were identified, and the most overrepresented categories were DNA binding (204 hits), protein binding (159 hits), and nucleus (118 hits).

### 2.10. MicroRNA Expression under Drought Stress

In order to check if our in silico analysis has support in wet-lab data, we performed differential expression analysis of identified miRNAs using small RNA-Seq data from a study that evaluated the effects of multiple drought cycles on *C. canephora* miRNA expression [24]. In our analysis, miR398 (Table S13, Supplementary File S2) was up-regulated in plants submitted to one drought-stress cycle. This result was also confirmed by stem-loop qPCR analysis [24], confirming that our approach was able to validate miRNA analysis in coffee.

### 2.11. Long Non-Coding RNAs

We identified 2384 high-confidence lncRNA loci, ranging from 200 to 6097 nucleotides (Table S17, Supplementary File S2). A total of 232 loci did not have transcriptional evidence (Table S17, Supplementary File S2). A total of 2531 high-confidence lncRNA regions were identified in the pseudomolecule “Chromosome 0”. The lncRNAs that were not assigned to any chromosome had sizes ranging from 200 to 8536 nucleotides (Table S24, Supplementary File S3).

Long non-coding RNAs are longer than 200 nucleotides and share some characteristics with messenger RNAs but have none or little protein-coding action [49]. Long non-coding RNAs are classified according to their function and position regarding protein-coding genes [8]. When overlapping protein-coding exons in the same direction, lncRNAs are classified as sense. When overlapping protein-coding exons in the opposite direction, they are antisense lncRNAs. If the lncRNA is inside an intron of a protein-coding gene, it is classified as an intronic lncRNA, and if it is between genes, it is classified as an intergenic lncRNA (lincRNA).

In *C. canephora*, 600 loci were identified as sense lncRNAs, 92 as antisense, 235 loci were overlapping another gene region, except exons, therefore, were classified as gene overlap lncRNAs, and 4220 did not overlap with genes.

## 3. Discussion

The results of the homology strategies show that ncRNAs searches require multiple combinations of computational strategies to detect the diversity of structure of the RNA families. In the case of snoRNA detection, the use of a specific tool for this kind of structure allowed a six-fold increase in the number of hits.

Several curation steps also improved the identification of miRNAs in the *C. canephora* genome. Due to the high number of false-positive miRNAs identified in plants, we chose to follow a strict set of rules to select high-confidence candidates in our prediction and previous studies. We noticed that more than half of miRNAs previously predicted in *C. canephora* could be false-positives, and even among the precursors that matched curation criteria, there were redundant sequences. miR-408 prediction and validation [24] did not match all curation criteria, but it was included in the final dataset due to its experimental validation. Thus, it is important to emphasize that wet-lab approaches will still be necessary to biologically validate miRNA families when “big data analysis” is not able to confirm their existence.

The application of clear and rigorous criteria is an important contribution to define miRNA families when data mostly relies on high throughput approaches, but it will probably underestimate miRNA families in genomes. Among the 72 miRNA precursors that matched curation criteria, 70 were variants of highly conserved plant miRNA families (detailed in Table S12, Supplementary File S2, and Supplementary File S3). The remaining two precursors belonged to family miR-157, involved in regulating vegetative phase change in *A. thaliana* [50], and family miR-3627, known for regulating plant metabolism and disease resistance [51].

At least four miRNA families (miR-160, miR-479, miR-827, and miR-1446), considered as highly conserved in plants, were previously identified in *C. canephora*, but they were excluded in our analysis. MiR-160 has been identified in two studies [22,24], but it was excluded because of the mature miRNA size and lack of expression. MiR-160 regulates the auxin response factors ARF10, -16, and -17 in plants [24,52] negatively. MiR-479 has been previously identified [23], but it was excluded because of the mature miRNA size. MiR-479 regulates plant metabolism and disease resistance, as in *V. vinifera* [51]. MiR-827 has been previously identified [22], but it was excluded in our analysis because the precursor size was greater than 300 nt and the unpaired position of mature miRNAs in the precursor. MiR-827 negatively regulates the expression of NITROGEN LIMITATION ADAPTATION (NLA), a ubiquitin E3 ligase gene (AT1G02860) in *A. thaliana* [53]. MiR-1446 has been previously identified [22], but it was excluded because of the lack of expression. MiR-1446 regulates disease resistance, as in *S. lycopersicum* [54].

In summary, we delivered here a consolidated and a highly-curated annotation for the ncRNA complement of *C. canephora* genome, unifying, revising, and expanding previous analyses.

## 4. Materials and Methods

### 4.1. Sequence Datasets

We downloaded the *C. canephora* genome in FASTA format at the Coffee Genome Hub [17] and ncRNAs sequences from Ensembl Plants version 34 [55]. Species available at Ensembl are described in Supplementary File S1. All Ensembl files were merged into one single FASTA file. Additional steps are illustrated in Figure S1, Supplementary File S1.

### 4.2. Similarity Search

The similarity search was done using BLASTN version 2.2.31 [56]. Ensembl Plants ncRNAs were used as a query against *C. canephora* genomic sequence with parameters word_size = 7, dust, and e-value = 10^−5^. These search results were filtered using an in-house Python script, maintaining sequences with query coverage and identity values over 90%. The script also compared the genomic positions of each sequence, considering strands. When overlapping cases were identified, the selection was made based on the highest query coverage, highest identity, lowest e-value, and highest bitscore value. Sequences with the size smaller than 60 nt were removed, and only tRNA, rRNA, miRNA, snRNA, and snoRNA hits were retrieved for further analyses.

### 4.3. Structural Search

ncRNAs were identified by structural search using Infernal version 1.1.2 [57] with 2582 family models from Rfam version 12.2 [58]. The cmsearch command was used with the cut_ga parameter. Results were then filtered using an in-house Python script, which compared the genomic positions of each sequence. In overlapping cases, candidates were selected with smaller e-values and higher bit-score values. Sequences smaller than 60 nt were excluded, and the sequences assigned as tRNA, rRNA, miRNA, snRNA, and snoRNA were retrieved. Additionally, ncRNA identification was performed using tools for specific ncRNAs classes.

### 4.4. Small Nuclear RNAs Filtering

All sequences identified as snRNAs from the similarity and structural searches were merged. Overlapping cases were manually analyzed based on the highest query coverage, highest identity, lowest e-value, and highest bitscore value. Most snRNAs can be categorized in spliceosomal U snRNAs, small nucleolar RNAs (snoRNA), and small Cajal body-specific RNAs (scaRNA), depending on their sub-nuclear localization, function, and structure [26]. We selected sequences from U snRNA spliceosomal families with sizes ranging between 60 and 220 nucleotides.

### 4.5. Small Nucleolar RNAs

We used SnoReport version 2.0 (UNB, Brasília, DF, Brazil) [59] with standard parameters to predict snoRNAs. We merged SnoReport2 results with previous approaches for snoRNA prediction (similarity and structural search). Overlapping cases were manually analyzed, and candidates were selected based on higher score values and lower e-values.

We defined as high-confidence C/D box snoRNA sequences with motifs RUGAUGA near the 5′-end for C box and CUGA near the 3′-end for D box. High-confidence H/ACA box snoRNAs were also identified for sequences with the motifs ACA box next 3′-end and an H box (ANANNA) located in the hinge region based on established criteria [29,60].

As most plant snoRNAs are organized in policistrons [29], we analyzed the genomic positions of identified snoRNA genes. Genomic regions harboring snoRNA genes within an interval smaller than 500 nt were considered snoRNA clusters [61].

### 4.6. Transfer RNAs

We used tRNAscan-SE version 1.3.1 (The Lowe Lab, University of California, Santa Cruz, CA, USA) [34] with standard parameters to identify tRNAs. These results were merged with data from similarity and structural searches. Sequences smaller than 70 nt and with more than 95 nt were excluded using a Python script, and the overlapping cases were manually analyzed. Final tRNA loci were defined based on higher score values and lower e-values. We also searched for tRNA^SeC^ genes on SecMarker pipeline [35], but none were found.

### 4.7. Ribosomal RNAs

rRNAs were searched using RNAmmer version 1.2 (DTU Bioinformatics, Lyngby, Denmark) [62] with standard parameters. RNAmmer results were merged with the structure and similarity searches. The overlapping cases were manually analyzed, and the final rRNA loci were defined based on higher score values and lower e-values.

### 4.8. MicroRNAs

All sequences identified as miRNA precursors from the similarity and structural searches were merged into one FASTA format file. This file was used as an input file on miRdup version 1.4 [41]. We trained miRdup with high-confidence plant miRNA precursor models and high-confidence plant miRNA mature models from miRBase release 21 [63], and then the sequences were confirmed as miRNA precursors. The positive cases were used as a new input file in miRdup to predict mature miRNAs. In this output, we applied Axtell and Meyers’ (2018) criteria to determine miRNA candidates. Additional steps are illustrated in Figure S2 and Supplementary File S1.

These criteria were also applied to the set of previously identified miRNAs in *C. canephora* [21,22,23,24] for curation. All sequences were compared with each other to find which ones were redundant. The miRNA family with the largest number of identified precursors in the *C. canephora* genome was selected for phylogenetic analysis.

Sequences were aligned using ClustalW in MEGA X software (Temple University, Philadelphia, PA, USA) [64] for molecular phylogenetic analyses with the following alignment parameters: gap opening 22.50; gap extension 0.83. A phylogenetic tree was inferred using the neighbor-joining method, and the sequence divergence was estimated using the p-distance method. The statistical reliability of the internal branches was assessed using 5000 bootstrap replicates.

*C. canephora* miRNA target genes were predicted using the *C. canephora* genes (CDS+UTR) sequence file from Coffee Genome Hub [17]. Targets were predicted using psRNATarget (Noble Research Institute, Ardmore, OK, USA) [65] with standard parameters. Results were classified as high-confidence candidates when the expectation value was lower than 2. The results were also classified according to their similarity with *A. thaliana* genes and the presence of protein domains. For this purpose, we used annotation data from PLAZA 4.0 [48] and InterPro 70 [66].

### 4.9. Long Non-Coding RNAs

For lncRNAs identification, we firstly analyzed a *C. canephora* transcriptome assembly [67] and *C. canephora* genes from Coffee Genome Hub [17]. First, sequences with more than 200 nucleotides were obtained using a Perl script from the RNAplonc tool version 1.0 (UTFPR, Cornélio Procópio, PR, Brazil) (200nt.pl) [68]. The remaining sequences were filtered using CD-HIT-EST [69] with 80% similarity cutoff. We then executed CPC version 2.0 [70] with standard parameters. Sequences classified as non-coding were considered as potential lncRNAs. This set of potential lncRNAs was analyzed against Uniref90 FASTA data [71] using the BLASTX version 2.2.31 [56] with an e-value of 10-5 and seg filter for low complexity regions. All ‘no-hit’ sequences were then considered lncRNAs. These lncRNA candidates had their coordinates determined in *C. canephora* genome using BLASTn version 2.2.31 (NCBI, Bethesda, MD, USA), with at least 95% of query coverage and identity, and applying in-house Python filters to select results and exclude redundancies.

Finally, we identified the coordinate intersection with other identified ncRNAs with BEDTools version 2.25.0 (University of Utah, Salt Lake City, UT, USA) [72], and the correspondent sequences were excluded. Additional steps are illustrated in Figure S3, Supplementary File S1. To classify lncRNA candidates, intersections were searched against the GFF3 data of *C. canephora* genes from Coffee Genome Hub [17] with BEDTools version 2.25.0 (University of Utah, Salt Lake City, UT, USA) [72]. The candidates were classified according to their distance from protein-coding genes: sense, for candidates that overlap exons on the same direction; antisense, for candidates that overlap exons on the opposite direction; gene overlap, for candidates overlapping with any another gene region except exons; no overlap, for candidates that do not overlap with genes.

### 4.10. Transcriptional Evidence Analysis

In order to have higher confidence in ncRNA loci, the transcriptional activity of ncRNAs was inferred by mapping small RNA-Seq data from three independent studies. We used data from NCBI accession GSE46617 [23], NCBI SRA accession PRJNA353111 [24], and we also performed a small RNA-seq run from *C. canephora* var C33 mature leaves (deposited in European Nucleotide Archive under accession PRJEB29660). The sRNA library was built and sequenced at the MGX platform (Institut de Génomique Fonctionnelle, Montpellier, France). The RNA library was constructed using the ‘RNA-seq sample prep’ kit (Illumina, San Diego, CA, USA). RNA was sequenced on a Hiseq 2500 (Illumina), in single-end, 50 bp sequencing. Raw data were analyzed using FastQC v0.11.7 (Babraham Bioinformatics, Cambridge, United Kingdom) [73], searching for low-quality reads and adapter sequences. Reads were then filtered using Trimmomatic version 0.36 (USADELLAB, Aachen, Germany) [74], removing reads smaller than 15 nt and a quality score below 30, along with adapters removal.

Filtered data was then mapped to the public sequence of the *C. canephora* genome [18], available at the Coffee Genome Hub [17] using STAR version 2.6.0 (Cold Spring Harbor Laboratory, Cold Spring Harbor, NY, USA) [75] with standard parameters. ncRNA loci with mapped reads from at least two independent small RNA-seq studies were considered as expressed ncRNAs, and regions with >10 mapping reads were considered as high-confidence ncRNA loci.

For miRNAs, we also performed differential expression analysis using mapped data [24] since it is the sole sRNA study that is a classic “treatment x control” experiment. A BED file with the microRNAs candidates coordinates was intersected against BAM files of mapping data from plants submitted to one (C1) or three cycles (C3) of drought stress to retrieve expression data. Analyses were carried out with BEDTools version 2.25.0 (University of Utah, Salt Lake City, UT, USA) [72] command intersect with -s parameter. Count files were used as input files in IDEAMEX [76] to perform differential expression analyses.

The results were the intersection of results from four validated Bioconductor packages—NOISeq, limma-voom, DESeq2, and edgeR [77]. MicroRNA candidates from intersected results file with FDR adjusted-*p*-value < 0.05 were considered as differentially expressed genes (DEG).

## 5. Conclusions

Although many studies have pointed out plant ncRNAs playing key roles in developmental [47,78] and regulatory processes [79,80], it is still uncommon to find studies identifying more than one or two ncRNA classes. Nevertheless, our analysis showed that, with several curation steps, it is possible to better assign most of the expected “housekeeping ncRNAs” and predict regulatory ncRNAs with high-confidence. Besides, merging predicted miRNAs from this study with results from previous studies, we obtained the first highly curated miRNA dataset for *C. canephora*.

Applying rigorous criteria based on the most recent plant miRNA annotation recommendation [15], we concluded that over 70% of *C. canephora* predicted precursor miRNAs were possibly false-positives. This was the most extensive genomic catalog of curated ncRNAs in the *Coffea* genus. The annotation of the *Coffea canephora* non-coding RNAs provided initial steps for a better understanding of the small RNA system in plants. Furthermore, it provided valuable research to establish curated non-coding RNA annotations for other plant genomes.

## Figures and Tables

**Table 1:** Comparative analysis of *C. canephora* genome features.

NcRNA Class (Genome Size)	*C. canephora* (~569 Mb)	*A. thaliana* (~135 Mb)	*C. sinensis* (~3.2 Gb)	*B. vulgaris* (~567 Mb)	*P. vulgaris* (~550 Mb)
tRNA	602	689	700	1043	712
rRNA	72	15	2860	231	145
miRNA	88	325	233	258	309
snRNA	115	82	223	121	165
snoRNA	1031	287	454	218	356
lncRNA	5064	3559	42,951	2546	1033
Total	6976	4957	47,421	4417	2720

## Supplementary Materials

The following are available online at https://www.mdpi.com/2311-553X/6/3/39/s1, Supplementary File S1: supplementary methods; Figures S1–S3; Tables S1–S6. Supplementary File S2: Tables S7–S17. Supplementary File S3: Tables S18–S24. Supplementary File S4: *C. canephora* microRNAs data. File Ccanephora_ncRNA.gff3 with *C. canephora* ncRNA coordinates. Part of these materials is available online at https://doi.org/10.7910/DVN/IUVZFV.
