# Supplementary material for: Genome-Wide Screening and Characterization of Non-Coding RNAs in Coffea canephora
Source: Noncoding RNA. 2020 Sep 11;6(3):39. doi: 10.3390/ncrna6030039 (PMC7549347; doi:10.3390/ncrna6030039)
Supplement: Supplementary file 1 [file ncrna-06-00039-s001.zip › Suppl_file_S1 (1).docx]

Supplementary Materials for

Genome-wide screening and characterization of non-coding RNAs in Coffea canephora

Samara Mireza Correia de Lemos^1^, Luiz Fernando Consalter Fonçatti^1^, Romain Guyot^3^, Alexandre Rossi Paschoal^1^, Douglas Silva Domingues^1,2^*

1 Department of Computer Science, Bioinformatics Graduation Program (PPGBIOINFO), Federal University of Technology - Paraná, Cornélio Procópio, 1640 Ave. Alberto Carazzai, PR, 86300-000, Brazil

2 Department of Botany, Institute of Biosciences, São Paulo State University, UNESP, Ave. 24-A 1515, CEP 13506-900, Rio Claro, SP, Brazil

3 Institut de Recherche pour le Développement (IRD), CIRAD, Univ. Montpellier, UMR IPME, Montpellier, France

* Corresponding author: Douglas Silva Domingues, Universidade Estadual Paulista (UNESP), Instituto de Biociências de Rio Claro, Avenida 24-A, 1515, CEP 13506-900, Rio Claro, SP, Brazil

This PDF file includes:

Materials and Methods

Supplementary Text

Figs. S1 to S3

Tables 1 to 7

Materials and Methods

Sequence Datasets

We downloaded the *C. canephora* genome in FASTA format at the Coffee Genome Hub, (Dereeper et al., 2014) and ncRNAs sequences from Ensembl Plants version 34 (Kersey et al., 2017). Species available at Ensembl are: *Aegilops tauschii, Amborella trichopoda, Arabidopsis thaliana, Brassica rapa, Chlamydomonas reinhardtii, Chondrus crispus, Cyanidioschyzon merolae, Galdieria sulphuraria, Glycine max, Hordeum vulgare, Leersia perrieri, Medicago truncatula, Musa acuminata, Oryza barthii, Oryza brachyantha, Oryza glaberrima, Oryza glumaepatula, Oryza indica, Oryza meridionalis, Oryza nivara, Oryza punctata, Oryza rufipogon, Oryza sativa, Prunus persica, Selaginella moellendorffii, Solanum lycopersicum, Solanum tuberosum, Sorghum bicolor, Triticum aestivum, Triticum urartu* and *Zea mays*. All Ensembl files were merged into one single FASTA file.


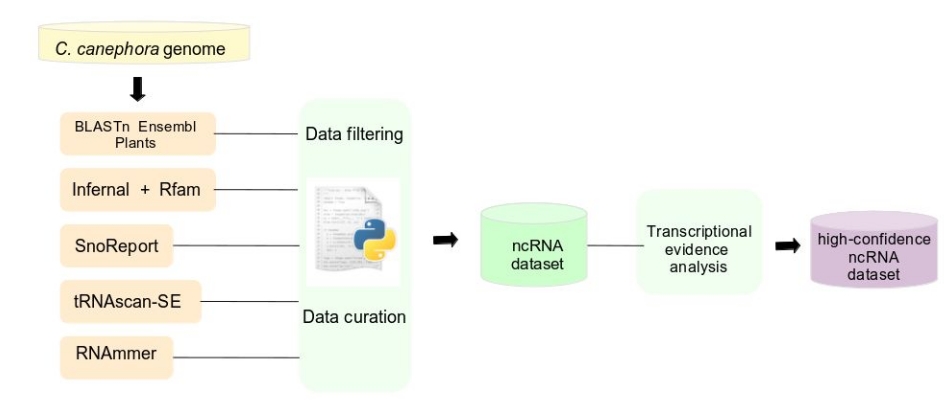


**Fig. S1:** Methods for ncRNAs analysis and selection.


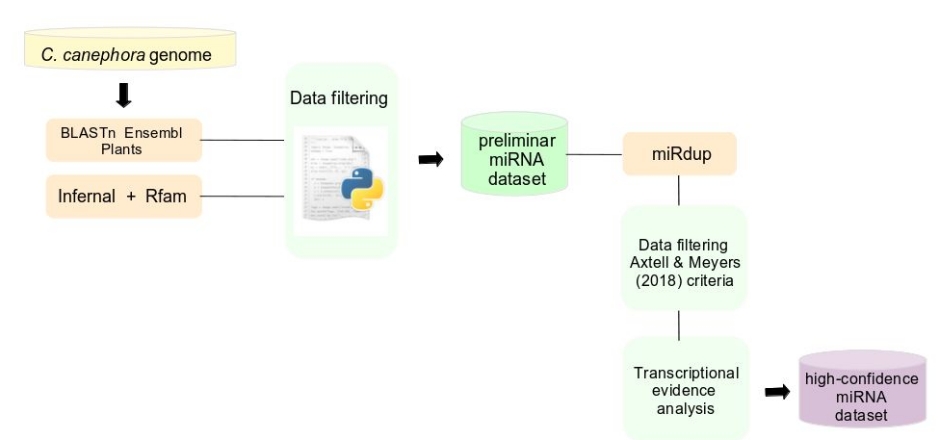


**Fig. S2:** Methods for miRNAs analysis and selection.


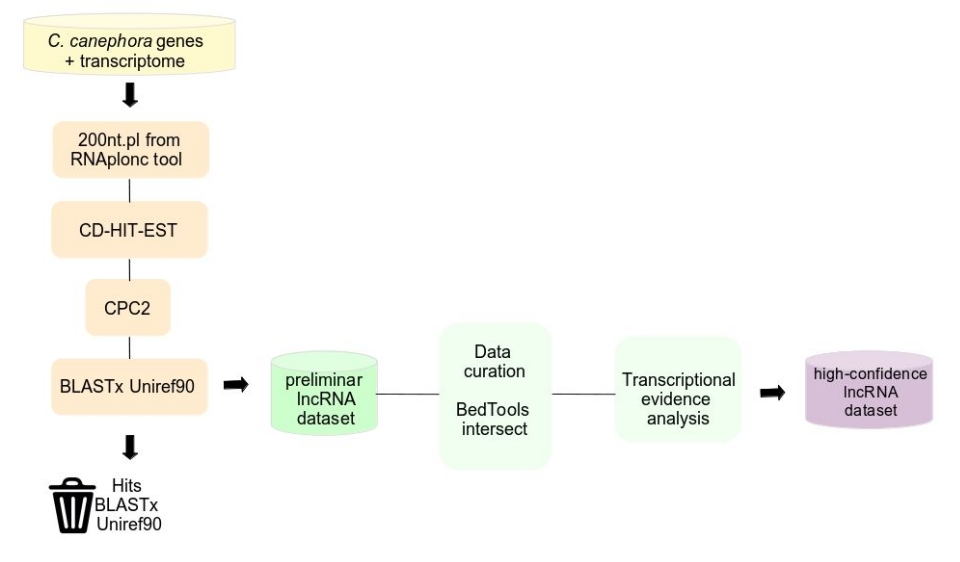


**Fig. S3:** Methods for lncRNAs analysis and selection.

**Table 1:** *Coffea canephora* genome data per chromosome. Size information, number of coding genes, coding genes/ Mb and TEs percentage were obtained in Denoeud et al. (2014).

| chr | size (Mb) | nº coding genes | coding genes/Mb | nº predicted ncRNAs | nº expressed ncRNAs | ncRNAs/Mb | %TEs |
| --- | --- | --- | --- | --- | --- | --- | --- |
| 1 | 38,2 | 2198 | 57,5 | 562 | 446 | 11,7 | 41,7 |
| 2 | 54,5 | 4000 | 73,4 | 763 | 626 | 11,5 | 35,2 |
| 3 | 32 | 1632 | 51 | 359 | 203 | 6,3 | 42,8 |
| 4 | 28,2 | 1727 | 61,2 | 401 | 317 | 11,2 | 39,7 |
| 5 | 29,1 | 1661 | 57,1 | 366 | 292 | 10 | 44,4 |
| 6 | 37,3 | 2839 | 76,1 | 489 | 413 | 11,1 | 38,4 |
| 7 | 29,8 | 2146 | 72 | 429 | 349 | 11,7 | 38 |
| 8 | 31,6 | 1718 | 54,4 | 400 | 328 | 10,4 | 41,6 |
| 9 | 22,3 | 1094 | 49,1 | 355 | 281 | 12,6 | 46,2 |
| 10 | 27,6 | 1653 | 59,9 | 360 | 300 | 10,9 | 40 |
| 11 | 33,5 | 1753 | 52,3 | 519 | 411 | 12,3 | 45,4 |
| 0 | 205,6 | 3603 | 17,5 | 3021 | 3014 | 14,6 | 64,7 |

**Table 2:** Number of expressed non-coding RNAs by chromosome and class in *Coffea canephora* genome.

| Number of ncRNAs identified by chromosome | | | | | | | |
| --- | --- | --- | --- | --- | --- | --- | --- |
| Chromosome (size) | tRNA | rRNA | miRNA | snRNA | snoRNA | lncRNA | Total |
| 1 (38,2 Mb) | 49 | 4 | 9 | 10 | 68 | 306 | 446 |
| 2 (54,5 Mb) | 91 | 1 | 23 | 11 | 104 | 396 | 626 |
| 3 (32 Mb) | 12 | 3 | 2 | 9 | 34 | 143 | 203 |
| 4 (28,2 Mb) | 28 | 7 | 10 | 9 | 85 | 178 | 317 |
| 5 (29,1 Mb) | 24 | 3 | 7 | 6 | 57 | 195 | 292 |
| 6 (37,3 Mb) | 45 | 1 | 9 | 9 | 73 | 276 | 413 |
| 7 (29,8 Mb) | 55 | 2 | 7 | 5 | 80 | 200 | 349 |
| 8 (31,6 Mb) | 45 | 3 | 5 | 12 | 60 | 203 | 328 |
| 9 (22,3 Mb) | 7 | 5 | 4 | 9 | 52 | 204 | 281 |
| 10 (27,6 Mb) | 33 | 1 | 3 | 7 | 81 | 175 | 300 |
| 11 (33,5 Mb) | 38 | 21 | 9 | 6 | 84 | 253 | 411 |
| Total | 427 | 51 | 88 | 93 | 778 | 2,529 | 3,966 |

**Table 3:** snoRNA families identified in *Coffea canephora* genome and their conservation among plants.

| snoRNA family | Conserved in Plants |
| --- | --- |
| snoR2, snoR18, snoR32_R81, snoR74, snoR96, U14a | highly conserved |
| snoR12, snoR16, SNOR19, snoR20a, snoR21, snoR24, snoR26, snoR31_Z110_Z27, snoR33, snoR35, snoR36, snoR44_J54, snoR60, snoR66, snoR69Y, snoR71/ R71, snoR77, snoR83, snoR86, snoR97, snoR99, snoR100, snoR103, snoR104, snoR109, snoR111, snoR126, snoR130, snoR135, snoR136, snoR137, snoR138, snoR145, U15b, U19, U30, U31b, Z102_R77, Z103, Z105, Z122, Z152, Z157, Z159, Z196, Z199, Z221_snoR21b, Z223, Z278, Z279_R105_R108 | conserved |
| SNOR75, snoR114, snoR117, snoR118, snoR128, snoR134, snoR143, snosnR60_Z15, U83, Z43, Z266 | - |

| **Table 4:** Comparison among the number of tRNA genes identified in seven plant genomes (adapted from Dohm et al., 2013; Mohanta & Bae, 2017). | | | | | | | |
| --- | --- | --- | --- | --- | --- | --- | --- |
|  | | | | | | | |
| **Amino acid** | ***C. canephora*** | ***B. vulgaris*** | ***A. thaliana*** | ***P. trichocarpa*** | ***V. vinifera*** | ***Z. mays*** | ***O. sativa*** |
| Ala | 32 | 92 | 33 | 43 | 63 | 122 | 44 |
| Arg | 42 | 73 | 39 | 44 | 34 | 85 | 59 |
| Asn | 20 | 37 | 19 | 22 | 22 | 49 | 32 |
| Asp | 26 | 46 | 28 | 33 | 25 | 42 | 30 |
| Cys | 19 | 23 | 17 | 14 | 11 | 28 | 18 |
| Gln | 18 | 27 | 19 | 22 | 19 | 37 | 41 |
| Glu | 28 | 40 | 27 | 35 | 32 | 57 | 31 |
| Gly | 43 | 88 | 43 | 49 | 35 | 54 | 47 |
| His | 15 | 26 | 12 | 13 | 14 | 30 | 26 |
| Ile | 18 | 59 | 25 | 30 | 21 | 79 | 23 |
| Leu | 42 | 76 | 45 | 58 | 51 | 74 | 58 |
| Lys | 23 | 52 | 33 | 34 | 26 | 349 | 32 |
| Met | 61 | 53 | 31 | 26 | 26 | 81 | 60 |
| Phe | 22 | 31 | 17 | 22 | 19 | 25 | 20 |
| Pro | 37 | 91 | 68 | 40 | 31 | 56 | 37 |
| Ser | 45 | 79 | 72 | 44 | 37 | 63 | 62 |
| Thr | 34 | 75 | 26 | 21 | 24 | 55 | 40 |
| Trp | 16 | 25 | 16 | 14 | 10 | 29 | 18 |
| Tyr | 16 | 72 | 83 | 18 | 19 | 26 | 22 |
| Val | 30 | 53 | 32 | 35 | 31 | 57 | 50 |
| Unknown | 15 | 23 | 1 | 3 | 8 | 12 | - |
| Total | 602 | 1141 | 686 | 620 | 558 | 1410 | 750 |

**Table 5:** Comparison among the number of tRNA genes identified in seven plant genomes (adapted from Dohm et al., 2013; Mohanta & Bae, 2017).

| **Amino acid** | **genes** | **C. canephora isoacceptors** | | | | | | **genes** | **O. sativa isoacceptors** | | | | |
| --- | --- | --- | --- | --- | --- | --- | --- | --- | --- | --- | --- | --- | --- |
| Alanine (Ala) | 32 | AGC (12) | CGC (3) | GGC (2) | TGC (15) |  |  | 44 | AGC (20) | CGC (13) | TGC (10) | GGC (1) |  |
| Arginine (Arg) | 42 | ACG (11) | CCG (5) | CCT (9) | TCG (4) | TCT (13) |  | 59 | ACG (24) | TCT (12) | CCT (11) | CCG (8) | TCG (4) |
| Asparagine (Asn) | 20 | ATT (1) | GTT (19) |  |  |  |  | 30 | GTT (30) |  |  |  |  |
| Aspartic acid (Asp) | 26 | GTC (26) |  |  |  |  |  | 32 | GTC (31) | ATC (1) |  |  |  |
| Cysteine (Cys) | 19 | GCA (19) |  |  |  |  |  | 18 | GCA (17) | ACA (1) |  |  |  |
| Glutamine (Gln) | 18 | CTG (6) | TTG (12) |  |  |  |  | 31 | TTG (21) | CTG (10) |  |  |  |
| Glutamic acid (Glu) | 28 | CTC (8) | TTC (20) |  |  |  |  | 41 | CTC (25) | TTC (16) |  |  |  |
| Glycine (Gly) | 43 | CCC (4) | GCC (29) | TCC (10) |  |  |  | 47 | GCC (28) | TCC (10) | CCC (9) |  |  |
| Histidine (His) | 15 | ATG (1) | GTG (14) |  |  |  |  | 26 | GTG (26) |  |  |  |  |
| Isoleucine (Ile) | 18 | AAT (14) | TAT (4) |  |  |  |  | 23 | AAT (18) | TAT (5) |  |  |  |
| Leucine (Leu) | 42 | AAG (6) | CAA (16) | CAG (7) | GAG (1) | TAA (5) | TAG (7) | 58 | CAA (19) | AAG (15) | CAG (10) | TAG (10) | TAA (4) |
| Lysine (Lys) | 23 | CTT (9) | TTT (14) |  |  |  |  | 32 | CTT (20) | TTT (12) |  |  |  |
| Methionine (Met) | 61 | CAT (61) |  |  |  |  |  | 60 | CAT (60) |  |  |  |  |
| Phenylalanine (Phe) | 22 | GAA (22) |  |  |  |  |  | 20 | GAA (20) |  |  |  |  |
| Proline (Pro) | 37 | AGG (8) | CGG (4) | GGG (1) | TGG (24) |  |  | 37 | AGG (14) | TGG (14) | CGG (9) |  |  |
| Serine (Ser) | 45 | AGA (9) | CGA (3) | GCT (12) | GGA (6) | TGA (14) | TCA (1) | 62 | GCT (20) | TGA (17) | AGA (12) | CGA (9) | GGA (4) |
| Threonine (Thr) | 34 | AGT (8) | CGT (2) | GGT (9) | TGT (15) |  |  | 40 | TGT (16) | AGT (11) | GGT (8) | CGT (5) |  |
| Tryptophan (Trp) | 16 | CCA (16) |  |  |  |  |  | 18 | CCA (18) |  |  |  |  |
| Tyrosine (Tyr) | 16 | GTA (16) |  |  |  |  |  | 22 | GTA (20) | ATA (2) |  |  |  |
| Valine (Val) | 30 | AAC (13) | CAC (8) | GAC (3) | TAC (6) |  |  | 50 | AAC (18) | GAC (17) | CAC (11) | TAC (4) |  |
| Undet. | 15 |  |  |  |  |  |  | - |  |  |  |  |  |
| Total | 602 |  |  |  |  |  |  | 750 |  |  |  |  |  |

**Table 6:** Curation of miRNAs from previous studies. We retrieved 494 precursors and 678 mature miRNAs.

| Criteria for miRNA annotation | Excluded precursors |
| --- | --- |
| Precursor miRNAs must have hairpins and be no larger than 300 nt | 14 |
| Precursor must have between one and three duplexes | 12 |
| Mature miRNA length between 20 and 22nt | 209 |
| Mature miRNAs without expression | 73 |
| Multiple criteria | 186 |
